# Nutrition in Pregnancy: Optimising Maternal Diet and Fetal Adaptations to Altered Nutrient Supply

**DOI:** 10.3390/nu8060342

**Published:** 2016-06-04

**Authors:** Janna L. Morrison, Timothy R. H. Regnault

**Affiliations:** 1 Early Origins of Adult Health Research Group, Sansom Institute for Health Research, University of South Australia, Adelaide 5001, Australia; 2 The Susan Vitali-Lovell Laboratories for Studies in Fetal Programming of Human Health Risks, Department of Obstetrics and Gynaecology and Physiology and Pharmacology, Western University, London, ON N6A-5C1, Canada; tim.regnault@uwo.ca

**Keywords:** maternal diet, fetal growth, postnatal health, adipose tissue, growth trajectory, macronutrients, micronutrient, fetal growth restriction, placenta

Maternal nutrition during pregnancy, and how this impacts placental and fetal growth and metabolism, is of considerable interest to women, their partners and their health care professionals. In developing countries, maternal undernutrition is a major factor contributing to adverse pregnancy outcomes. Conversely, with the increased prevalence of high calorie diets and resulting overweight and obesity issues in developed countries, the impact of this overnutrition situation upon pregnancy outcome is highlighted as a contributing factor for adverse metabolic outcomes in offspring later in life. Further, while low or excessive food intake *per se* is an important aspect of pregnancy development, the specific role that the placenta plays in nutrient metabolism and overall nutrient supply to the fetus in situations of undernutrion, overnutrition or poor diet composition is still poorly defined. Both epidemiology and animal studies now highlight that undernutrition, overnutrition, and diet composition negatively impact fetoplacental growth and metabolic patterns, having adverse later life metabolic effects for the offspring. This issue aims to highlight new research in a number of these abovementioned areas across the early life course.

A great deal of data now highlights the periconceptional period as a critical period upon which insults may generate later life physiological and metabolic changes in the resulting offspring. In the review submitted by Padhee and colleagues [1], the procedures of ARTs are examined, specifically in terms of how common procedures associated with the handling and preparation of gametes and embryos may impact later life metabolism, particularly impacting offspring cardiometabolic health. These later life defective metabolic effects are also understood to be established during pregnancy. In surveying preconceptional women, pregnant and lactating women and women of reproductive age, Cuervo *et al.* report that these groups are not appropriately consuming foods for their physiological status, based upon the Spanish dietary guidelines and highlight a real need for improved education and community outreach programs to these groups of women to ensure adequate maternal and thus fetal nutrition [2].

Poor maternal nutritional intake after the periconceptional period during pregnancy can also negatively impact fetal genetic growth trajectory and can result in fetal growth restriction. Vonnahme *et al*. [3], describe the effects of maternal undernutrition on vascularity of nutrient transferring tissue during different stages of pregnancy. In addition to maternal nutrient supply, the effectiveness of the placenta in transporting nutrients and oxygen to the fetus is important in determining fetal growth. A range of adaptations to placental development occur when the fetus is growth restricted and these are described by Zhang *et al.* [4]. Regardless of the cause of low birth weight, Zheng *et al.* show a relationship between the placental microbiome and fetal growth [5]. Zohdi *et al*. describe the effects of maternal protein restriction during pregnancy on fetal development that increase the risk of cardiovascular disease later in life [6]. Davis *et al.* illustrate the importance of the adrenal gland in the fetal adaptation for placental insufficiency, highlighting the important role of norepinephrine in regulating fetal growth but not pancreatic mass in the growth restricted fetus [7]. Wood-Bradley and team provide a review of the literature surrounding the potential mechanisms by which maternal nutrition (focusing on malnutrition due to protein restriction, micronutrient restriction and excessive fat intake) influences offspring kidney development and thereby function in later life [8]. In the same light, Blumfield *et al*. detail evidence that a maternal diet during pregnancy that is low in protein is related to higher systolic blood pressure in childhood [9]. Furthermore, Colon-Ramos and colleagues investigated the potential association between maternal dietary patterns during pregnancy and birth outcomes in a diverse population with a historically high burden of low birth weight and other adverse birth outcomes [10].

Experiences in the perinatal period also play a key role in defining how offspring respond to stress(es) in postnatal life. To this point, Tsuduki and colleagues report upon the impact a high fat diet during mouse lactation, where it appears to increase the susceptibility of later life obesity induced through postnatal social stress [11]. This paper highlights the importance of understanding how an early life environment predisposes offspring to potential detrimental responses to postnatal adverse situations. In a review by Dunlop *et al*., the impact of fetal growth restriction on postnatal metabolism in skeletal muscle, but also the effect of a “second hit”, such as a Western diet in postnatal life, is presented [12].

While meeting dietary guidelines is important, overall maternal health status also plays a pivotal role in determining fetal nutrient supply. In situations of maternal disease, such as infection with human immunodeficiency virus (HIV), the ability of the mother to consume sufficient substrates to maintain herself and meet fetal demands is often compromised. In situations of HIV, resting energy expenditure is increased and the disease may limit dietary intake and reduce nutrient absorption, in addition to influencing the progression of HIV disease as reported by Ramlal and colleagues [13]. Their study described typical diets of HIV-infected, pregnant Malawian women and highlighted that poor quality maternal diets should be enhanced to meet demands of this particular group of pregnant women, vulnerable to both HIV and malnutrition.

While deficiencies in nutrition during pregnancy can result in adverse offspring outcomes, once pregnant, maternal weight gain during and after pregnancy are critical issues for maternal and fetal health as well. In the pilot RCT report lead by Martin *et al*., a cohort of women were recruited with the aim of reducing postpartum weight retention and improving breastfeeding outcomes [14]. The findings indicate that the approach reported is feasible and acceptable to pregnant women and that the methodology, including the collection of blood for biomarker assessment, could be adapted based on qualitative feedback to a larger, adequately powered RCT. Assessing maternal body composition, as part of monitoring maternal well-being, prior to and during pregnancy is critical to estimate the requirements for dietary energy during gestation and when investigating relationships between maternal nutritional status and offspring development. Forsum and co-workers investigate the possibility of estimating body density and the use of a two-component model (2CM) to calculate total body fat concluding it may present a new clinically appropriate methodology [15].

Many nutritional studies in pregnancy have focussed on the impact of changes in total or macronutrient intake. This current issue features several studies that expand our knowledge regarding nutrient uptake during pregnancy, but have focused on changes in micronutrients during pregnancy. Grieger and Clifton, provide updated evidence from epidemiological and RCTs on the impact of dietary and supplemental intakes of omega-3 long-chain polyunsaturated fatty acids, zinc, folate, iron, calcium, and vitamin D, as well as dietary patterns, on infant birth weight [16]. Additionally, in studying maternal intakes of polyunsaturated fatty acids (PUFAs), Bascuñán *et al.* report a Chilean study that highlights the need for new strategies to improve *n*-3 PUFA intake throughout pregnancy and breastfeeding periods and the need to develop dietary interventions to improve the quality of consumed foods with particular emphasis on *n*-3 PUFA for adequate fetal development [17]. Fish intake during pregnancy is recognized as an important source of PUFAs. Starling and co-workers present a systematic review of fish intake during pregnancy and fetal neurodevelopment [18]. The review covers approximately a 14 year period of publications between January 2000 and March 2014 involving over 270 papers, of which only eight were selected for a qualitative comparison of study findings.

Deficiencies in a range of micronutrients in low vs middle income countries that may act through epigenetic mechanisms to influence fetal development and risk of chronic disease in adult life are identified by Darnton-Hill *et al.* [19]. They also discuss supplementation programs. One particular micronutrient that is important for sulphonation of steroids and hormones is sulphate. Dawson *et al.* describe the requirements for sulphate during pregnancy, the consequences of reduced sulphonation capacity and the use of animal models to adequately understand the role of sulphate in human pregnancy [20]. Folic acid and Vitamin B12, are crucial factors for metabolic pathways, and have been extensively studied and demonstrated to play important roles in preventing the development of neural tube defects (NTDs). Wang *et al*. present data that in a local Chinese population consumption of non-staple foods such as milk, fresh fruits, and nuts were associated with decreasing NTDs risk in offspring [21]. Further independent roles for folate and Vitamin B12 deficiency amongst pregnant women are presented in this issue. The relationship between maternal Vitamin B12 neonatal HDL is presented by Adaikalakotwewari *et al*. [22], and folate deficiency resulting in birth defects is highlighted by Li *et al.*, who present a mouse model to provide evidence that folate deficiency can impair decidual angiogenesis [23].

The importance of adequate Vitamin D in women of reproductive age and its role in fetal development is of great interest and importance. A review of calcitrol biosynthesis during pregnancy, particularly by the placenta is presented by Olmos-Ortiz *et al.* [24]. Additionally, Choi *et al.* describe the high prevalence of Vitamin D deficiency in Korean women during pregnancy [25], particularly in the winter, while Yu *et al.* report the cord blood Vitamin D in babies born in Shanghai [26]. Finally regarding Vitamin D, the impact of sun exposure and Vitamin D supplementation on achieving appropriate Vitamin D status in women whom are breastfeeding is explored by Dawodu and colleagues [27].

In this issue, several new studies highlighted the importance of diet intake and composition upon maternal and fetal well-being parameters in human population and animal studies. Many of these studies show that deficiencies in consumption/delivery of components (e.g., protein, vitamins, PUFAs) of a diet can lead to adverse fetal/offspring development and detail how consumption of certain foods may have beneficial effects on fetal/offspring growth and development. We hope that the articles contained within this issue, and the material they reference and describe, are of interest to women, their partners and their health care professionals in promoting continual and informed dialogue about nutrition in pregnancy.
